# Linked sensitization by memory CD4^+^ T cells prevents costimulation blockade–induced transplantation tolerance

**DOI:** 10.1172/jci.insight.159205

**Published:** 2022-06-08

**Authors:** Michael S. Andrade, James S. Young, Jared M. Pollard, Dengping Yin, Maria-Luisa Alegre, Anita S. Chong

**Affiliations:** 1 Section of Transplantation, Department of Surgery, and; 2 Section of Rheumatology, Department of Medicine, University of Chicago, Chicago, Illinois, USA.

**Keywords:** Immunology, Transplantation, Tolerance

## Abstract

Dominant infectious tolerance explains how brief tolerance-inducing therapies result in lifelong tolerance to donor antigens and “linked” third-party antigens, while recipient sensitization and ensuing immunological memory prevent the successful induction of transplant tolerance. In this study, we juxtapose these 2 concepts to test whether mechanisms of dominant infectious tolerance can control a limited repertoire of memory T and B cells. We show that sensitization to a single donor antigen is sufficient to prevent stable transplant tolerance, rendering it unstable. Mechanistic studies revealed that recall antibody responses and memory CD8^+^ T cell expansion were initially controlled, but memory CD4^+^Foxp3^–^ T cell (Tconv) responses were not. Remarkably, naive donor-specific Tconvs at tolerance induction also acquired a resistance to tolerance, proliferating and acquiring a phenotype similar to memory Tconvs. This phenomenon of “linked sensitization” underscores the challenges of reprogramming a primed immune response toward tolerance and identifies a potential therapeutic checkpoint for synergizing with costimulation blockade to achieve transplant tolerance in the clinic.

## Introduction

Transplantation tolerance can facilitate long-term allograft acceptance while avoiding the need for lifelong immunosuppression and its associated problems. Seminal studies by Medawar and colleagues provided proof-of-principle evidence that tolerance can be achieved by exposing fetal mice to allogeneic cells, and over the past decade, operational clinical transplantation tolerance has been shown to be achievable through hematopoietic stem cell transplantation or weaning of conventional immunosuppression (1–9). Successful immunosuppression weaning is achievable in 22%–62.5% of selected liver transplant recipients but is infrequently successful for kidney transplants (1, 10–13). Importantly, even in the most highly selected liver transplant recipients, 40%–50% fail to achieve tolerance and instead develop graft rejection during or after weaning of immunosuppression (10, 11, 14). Likewise, an investigation into the natural history of 27 cases of long-term operational tolerance to kidney allografts (6) revealed that approximately 30% presented with slow deterioration of graft function. Collectively, these observations suggest that while operational tolerance can be achieved and persist as a robust phenomenon, the majority of transplant recipients fail to achieve stable tolerance, and a subset of long-term operationally tolerant transplant recipients eventually experience graft loss. Thus, preclinical investigations are needed to identify biomarkers predicting stable versus unstable or failed tolerance and to develop new strategies for overcoming barriers to long-term stable tolerance.

Interest in costimulation blockade (CoB) via anti-CD154 as a means of inducing transplantation tolerance was prompted by preclinical data from rodent and nonhuman primate studies showing prolonged allograft survival after cessation of treatment (15–18). However, longer-term follow-up studies with nonhuman primate recipients revealed rejection after 10 months after treatment cessation (19). Subsequent studies identified high frequencies of memory alloreactive T cells as a key barrier to the successful induction of tolerance, and experimental data from mouse models showed that adoptively transferred memory CD4^+^ or CD8^+^ T cells are resistant to the tolerogenic effects of CoB (20–27). Despite successful restraint of memory T cells with additional immunosuppression, rejection typically ensues upon cessation of immunosuppression (28). These observations suggest that memory T cells resist programming into the cell-intrinsic dysfunctional states naive T cells attain. This behavior in memory T cells may be explained by their reduced dependence on CD28:B7 and CD40:CD154 costimulation, preferential migration to inflamed tissue, and relative resistance to CD4^+^Foxp3^+^ regulatory T cells (Tregs) (27, 29–33).

Infectious tolerance is defined as the ability of adoptively transferred tolerant cells to induce donor-specific tolerance in naive recipients, whereas linked suppression is defined as the ability for tolerant mice to develop tolerance to new alloantigens if those antigens are initially introduced as F1 grafts coexpressing tolerized and nontolerized antigens (34–36). Peripheral transplantation tolerance capable of mediating infectious tolerance and linked suppression is induced in naive recipients of allografts, following transient treatment with anti-CD4, anti-CD8, or CoB (34, 35, 37–43). In these models, Foxp3^+^ Tregs have been reported to play a dominant role in the induction and maintenance of transplant tolerance by exerting regulatory microenvironments in lymphoid and allograft tissues and by dampening alloreactive T cells at multiple stages, including terminal differentiation of effector T cells (39, 44–46). These observations raise the possibility that these potent mechanisms of infectious tolerance and linked suppression may also be able to restrain memory T and B cell responses, ultimately reprogramming them toward cell-intrinsic dysfunction.

We developed a transplant model where C57BL/6 (B6) recipients are presensitized to a single donor antigen, 2W-OVA, and then transplanted with 2W-OVA.BALB/c × B6 (2W-OVA.F1) heart allografts under cover of a tolerance-inducing treatment of anti-CD154 plus donor spleen cells (TolRx) (Supplemental Figure 1; supplemental material available online with this article; https://doi.org/10.1172/jci.insight.159205DS1). We reasoned that in these recipients, the large repertoire of naive BALB/c-specific T cells would acquire the ability to mediate infectious tolerance/linked suppression and thus allow us to test whether these tolerance mechanisms are able to dominantly control a much smaller population of 2W-OVA–specific memory T and B cells. We observed that as an unstable state of tolerance developed, recall B cell responses and the expansion of memory CD8^+^ T cells were controlled, but memory CD4^+^Foxp3^–^ T cell (Tconv) responses were not. Furthermore, presensitization to a single donor antigen led to “linked sensitization” whereby naive CD4^+^ T cells of additional donor specificities became resistant to TolRx-induced tolerance. That linked sensitization eventually dominates over infectious tolerance, and linked suppression underscores a new and potent barrier to the induction of tolerance in recipients harboring even a limited repertoire of donor-specific memory Tconvs.

## Results

### Presensitization to a single donor antigen results in unstable transplantation tolerance.

**Figure 1:**
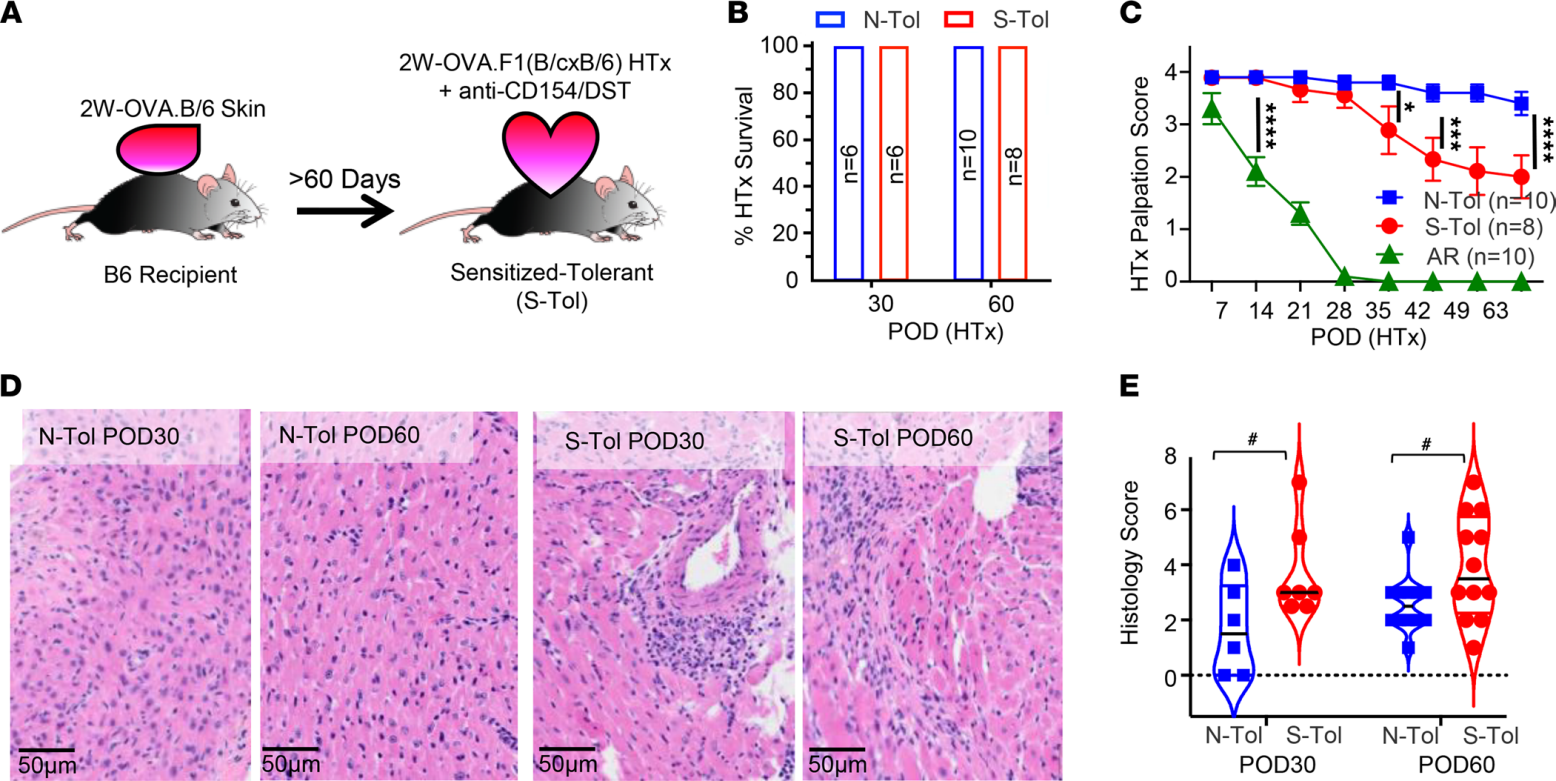
Presensitization to a single donor antigen results in unstable transplantation tolerance. (**A**) Experimental design. B6 female mice were sensitized to 2W-OVA with skin grafts from female 2W-OVA.B6 donors. After 60–90 days, sensitized mice or age-matched naive control animals received 2W-OVA.F1 heart allografts and anti-CD154 + donor splenocyte transfusion (DST) to induce tolerance. (**B**) Graft survival on postoperative day (POD) 30 and POD 60 (*n* = 6–10 mice per group). (**C**) Graft palpation scores of transplanted allograft in naive-tolerant (N-Tol), 2W-OVA skin sensitized+tolerant (S-Tol), and acute rejecting (AR) mice on HTx POD 60. All experiments were repeated at least 3 times (*n* = 8–10 mice per group). (**D**) Representative histology at 40× original magnification for allografts from N-Tol and S-Tol mice on POD 30 and POD 60. (**E**) Histology scores from N-Tol and S-Tol on POD 30 and POD 60. At least *n* = 6 sections per group were analyzed, and data are presented as violin plots with the median indicated as black bars. Statistical significance was assessed by 2-way ANOVA and Tukey’s multiple comparisons test *P < 0.05, ***P < 0.001, ****P < 0.0001, or Mann-Whitney test ^#^*P* < 0.05.

We hypothesized that to achieve stable transplantation tolerance in presensitized recipients, donor-specific memory T cells must either be programmed to dysfunctional states comparable to naive T cells or, at minimum, be stably suppressed by mechanisms of infectious tolerance and linked suppression. We used a model in which B6 mice were sensitized to a single fusion protein, 2W-OVA, via the acute rejection of 2W-OVA.B6 skin grafts (Figure 1A). At 60–90 days after skin sensitization, these mice were used as recipients of 2W-OVA.F1 heart transplants (HTx) plus TolRx and are referred to as sensitized tolerant (S-Tol) recipients. This experimental model enabled us to test whether the larger repertoire of naive anti-BALB/c T cells can exert dominant tolerance mechanisms to sustainably control the limited repertoire of 2W-OVA–specific memory T and B cell responses.

In naive recipients of 2W-OVA.F1 HTx (naive tolerant, or N-Tol, mice), TolRx treatment induced stable graft acceptance, with sustained graft palpation scores over the 60-day observation period (Figure 1, B and C). In S-Tol mice, none of the 2W-OVA.F1 HTx were fully rejected at POD 60, but a significant decay in heart palpation score was detected starting on approximately POD 35. Damage to cardiac tissue was confirmed via histological analysis revealing increased cellular infiltrate and tissue damage in grafts from S-Tol compared with N-Tol recipients (Figure 1, D and E). Collectively, these data suggest that sensitization to a single donor protein is sufficient to prevent the induction of stable transplantation tolerance, and a state of unstable tolerance ensued.

### Unstable transplantation tolerance is not driven by antibody-mediated rejection or the accumulation of memory OVA:K^b^ CD8^+^ T cells.

**Figure 2:**
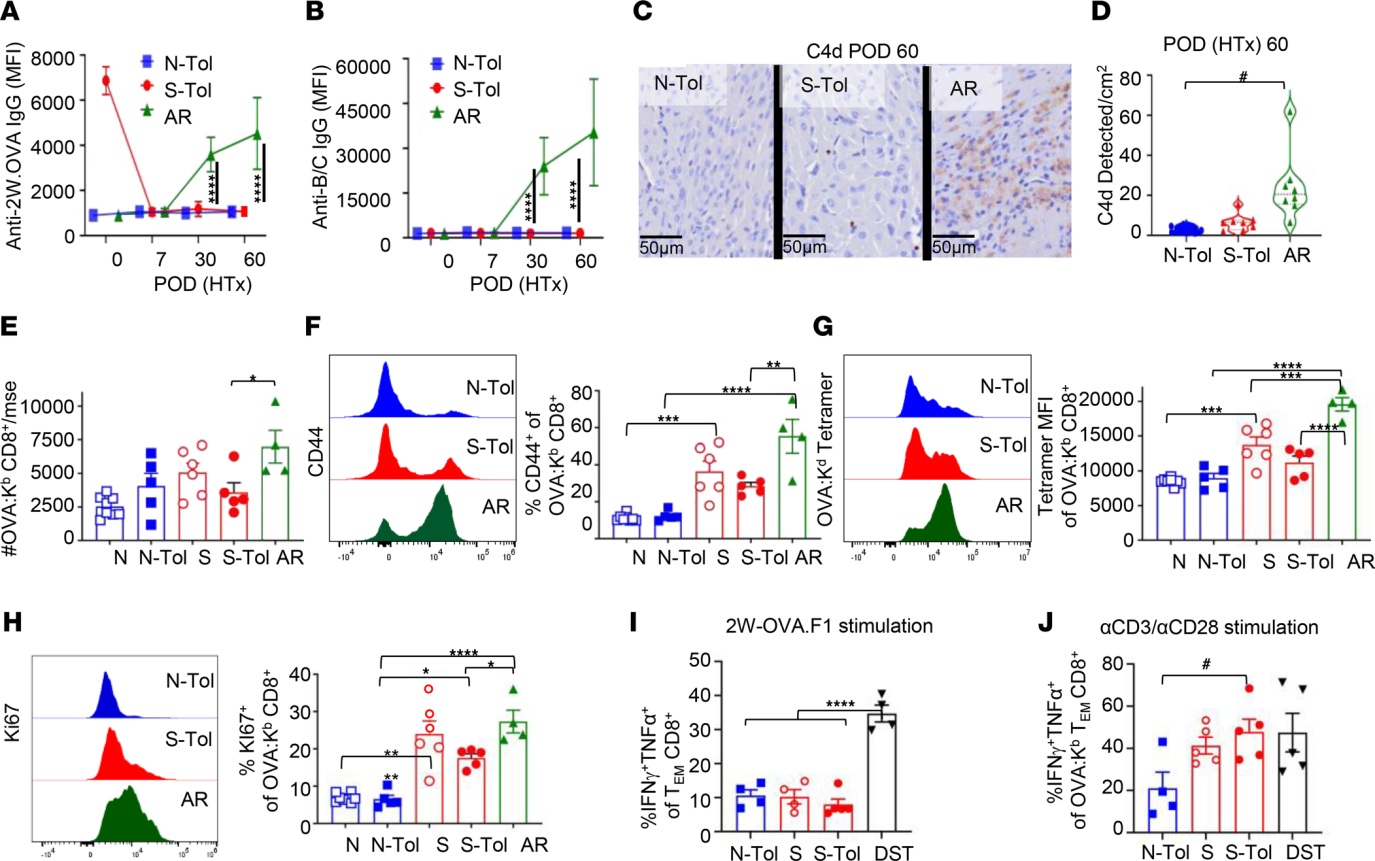
Unstable tolerance is not associated with the accumulation of memory OVA:K^b^ CD8^+^ T cells. (**A**) Serum anti–2W-OVA IgG and (**B**) anti-BALB/c IgG on HTx POD 0, 7, 30, and 60 was quantified on 2W-OVA.B6 lymphocytes and BALB/c lymphocytes, respectively. Mean fluorescence intensity (MFI) of IgG binding on CD19^–^ lymphocytes is presented as mean ± standard deviation (STDEV) (*n* = 4–5 mice per group). (**C**) Representative immunohistochemistry staining at 40× original magnification for C4d for N-Tol, S-Tol, and AR recipients on POD 60. (**D**) C4d quantification per cm^2^ on POD 60 was conducted on a total of *n* = 8 sections per group using QuPath automatic cell detection software. (**E**) Total number of OVA:K^b^ CD8^+^ T cells recovered from spleen and lymph nodes/mouse of naive (N), N-Tol, sensitized (S), S-Tol, and AR mice on POD 60. (**F**) Representative histograms and percentage of CD44^+^ of OVA:K^b^ CD8^+^ T cells. (**G**) MFI of OVA:K^b^ tetramer binding and (**H**) percentage of Ki67^hi^ of OVA:K^b^ CD8^+^ T cells. (**I** and **J**) Percentage of IFN-γ^+^ and TNF-α^+^ effector memory (CD44^+^CD62L^–^) OVA:K^b^ CD8^+^T cells (on POD 60) stimulated in vitro with 2W-OVA.F1 T cell–depleted splenocytes (**I**) or αCD3/αCD28 stimulation (**J**). Each symbol represents a single mouse, and each experiment was repeated 2–3 times (*n* = 4–8 mice per group). Data are presented as mean ± STDEV, and statistical significance was assessed by 1-way ANOVA and Tukey’s or Dunnett’s multiple-comparison test *P < 0.05, **P < 0.005, ***P < 0.001, ****P < 0.0001, or Mann-Whitney test ^#^*P* < 0.05.

In the clinic, presensitization resulting in a detectable donor-specific antibody response strongly predisposes the recipient to antibody-mediated graft rejection (30, 47, 48), and the presence of donor-specific antibodies and memory B cells at the time of HTx prevents CoB-induced tolerance in preclinical models (49). Thus, we tested whether recall 2W-OVA IgG responses were elicited in unstable tolerance. Presensitization with 2W-OVA skin grafts resulted in increased anti–2W-OVA IgG (Figure 2A), but they rapidly diminished to undetectable levels after HTx+TolRx in S-Tol recipients. Furthermore, anti-BALB/c IgG also remained low in S-Tol recipients (Figure 2B). Nevertheless, the presence of circulating anti–2W-OVA IgG detected more than 60 days after 2W-OVA skin transplantation raised the possibility that these IgGs may be causing antibody-mediated rejection (29, 30). We therefore probed for the presence of complement protein C4d in heart allografts (Figure 2, C and D). While C4d deposition was detected in acutely rejected HTx at POD 60, minimal C4d was observed in the heart grafts from N-Tol or S-Tol mice. These data suggest that recall anti–2W-OVA IgG responses are controlled and that there is a lack of evidence of active antibody-mediated rejection during unstable tolerance.

Past investigations into the basis for resistance to CoB-induced transplantation tolerance revealed a role for either memory CD4^+^ or CD8^+^ T cells, with memory CD8^+^ T cells being more effective at preventing CoB-induced tolerance compared with memory CD4^+^ T cells (21). We quantified OVA:K^b^ tetramer-binding CD8^+^ T cells (Supplemental Figure 2A) to show that OVA:K^b^ CD8^+^ T cells markedly expanded following acute rejection of HTx (POD 60). However, no significant expansion was observed in S-Tol recipients on D60 post-HTx, compared to sensitized recipients (Figure 2E). To address the possibility that memory OVA:K^b^ CD8^+^ T cells in S-Tol recipients may have expanded and then contracted, we verified that there was no significant increase in the percentage of OVA:K^b^ CD8^+^ T cells that expressed CD44 or Ki67, and they had not undergone avidity maturation (defined by increased OVA:K^b^ tetramer binding) in S-Tol compared to skin-sensitized recipients (Figure 2, F–H) (50). Furthermore, there was no increased accumulation of CD8^+^ T cells in the allografts with unstable tolerance compared to stable tolerance (Supplemental Figure 3, A–C). Collectively, these data indicate that the expansion of memory CD8^+^ T cell responses was inhibited on POD 60 post-HTx as unstable tolerance developed.

Reduced cytokine production by donor-specific T cells is another characteristic feature of transplantation tolerance (51, 52). Indeed, CD8^+^ T cells from N-Tol and S-Tol mice stimulated with 2W-OVA.F1 splenocytes exhibited comparably low percentages of dual IFN-γ and TNF-α production (Figure 2I). To address whether memory 2W-OVA CD8^+^ T cells had lost their ability to produce IFN-γ and TNF-α entirely, T cells from N-Tol or S-Tol mice were stimulated with anti-CD3/CD28, and intracellular IFN-γ and TNF-α production by OVA:K^b^ CD8^+^ T cells was determined (Supplemental Figure 2B). The percentage of dual IFN-γ^+^TNF-α^+^ OVA:K^b^ CD8^+^ T cells from S-Tol and skin-sensitized mice was comparable and significantly higher than from N-Tol recipients (Figure 2J). These observations suggest that despite persistent exposure to alloantigen in unstable tolerance, memory OVA-specific CD8^+^ T cells did not acquire an exhausted state that is marked by cell-intrinsic loss of cytokine production (53–55). Indeed, while memory OVA:K^b^ CD8^+^ T cell responses were inhibited on HTx POD 30 and 60, they significantly accumulated by HTx POD 90 (Supplemental Figure 5, A–C).

### Unstable tolerance is associated with accumulation of memory 2W:I-A^b^ CD4^+^ Tconvs.

**Figure 3:**
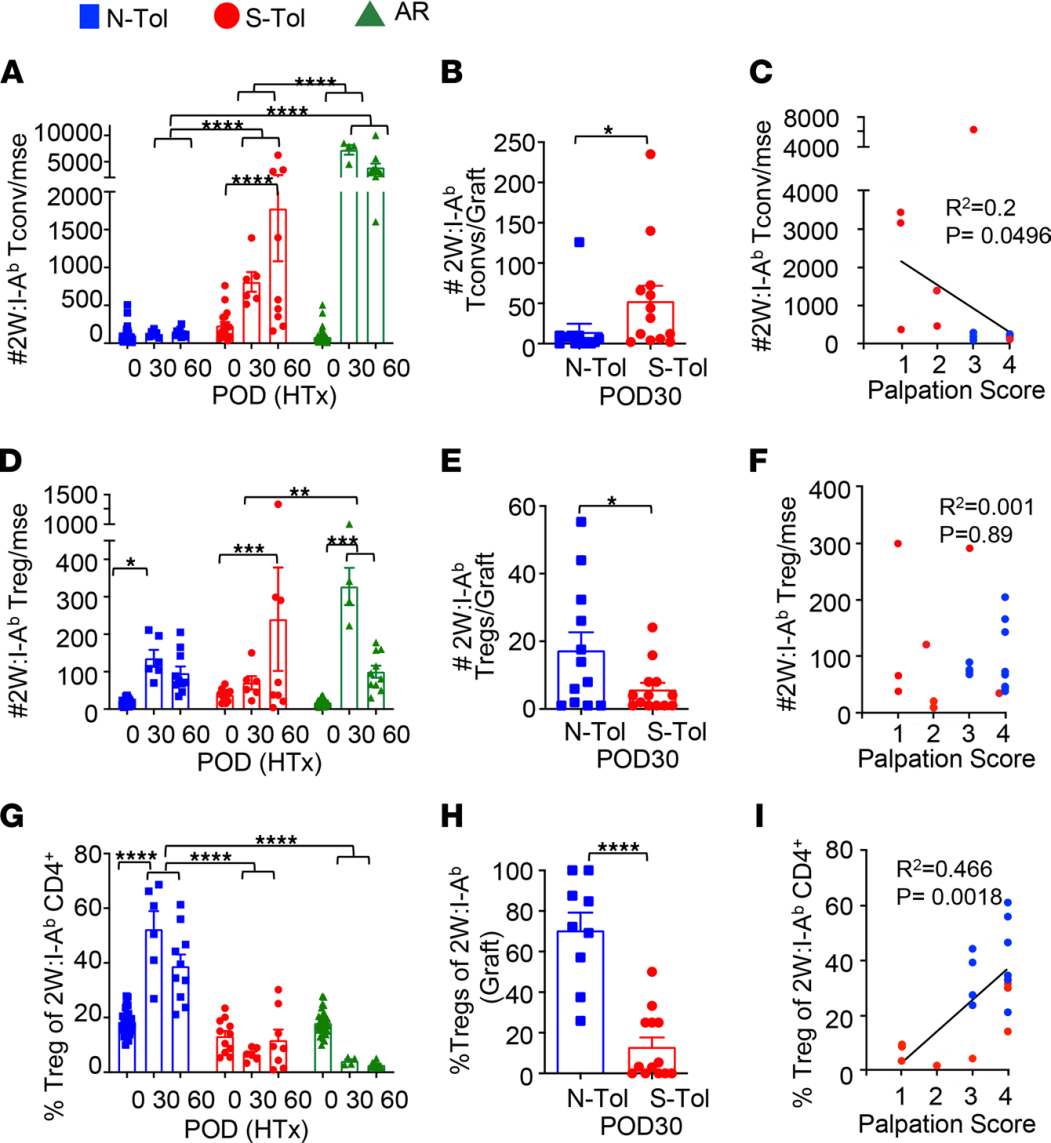
Unstable tolerance is associated with the accumulation of memory 2W:I-A^b^ Tconvs and reduced expansion of memory 2W:I-A^b^ Tregs. (**A** and **B**) Number of 2W:I-A^b^ CD4^+^ Tconvs recovered per mouse (at least *n* = 6 per group) from (**A**) spleen and lymph nodes on HTx POD 0, 30, and 60 or (**B**) transplanted allograft POD 30. (**C**) Number of 2W:I-A^b^ CD4^+^ Tconvs recovered from spleen and lymph nodes/mouse inversely correlates with graft palpation scores assessed on POD 60. (**D** and **E**) Number of 2W:I-A^b^ Tregs recovered per mouse from (**D**) spleen and lymph nodes on HTx POD 0, 30, and 60 or (**E**) transplanted allograft POD 30. (**F**) Number of 2W:I-A^b^ Tregs recovered from the spleen and lymph nodes/mouse does not correlate with graft palpation scores at POD 60. (**G** and **H**) Percentage Tregs of 2W:I-A^b^ CD4^+^ from (**G**) the spleen and lymph nodes on POD 0, 30, and 60 or (**H**) transplanted allograft POD 30. (**I**) Percentage of Tregs of 2W:I-A^b^ T cells recovered from the spleen and lymph nodes/mouse correlates with graft palpation scores at POD 60. Each symbol represents a single mouse, and each experiment was repeated 2–3 times (*n* = 4–5 mice per group). Data are presented as mean ± STDEV, and statistical significance was assessed by 2-way ANOVA and Tukey’s multiple comparisons test (**A**, **D**, and **G**) or Welch’s *t* test (**B**, **E**, and **H**) *P < 0.05, **P < 0.01, ***P < 0.001, ****P < 0.0001, or by simple linear correlation (**C**, **F**, and **I**).

Since memory CD8^+^ T cells and B cell responses were largely controlled during the development of unstable tolerance, we next tested whether memory 2W:I-A^b^ CD4^+^ T cells were resistant to TolRx-induced infectious tolerance. We quantified 2W-reactive CD4^+^ T cells using 2W:I-A^b^ tetramers (Supplemental Figure 2C) to show that the expansion of 2W:I-A^b^ Tconvs was inhibited in N-Tol mice, whereas a significant accumulation of 2W:I-A^b^ Tconvs was observed as early as POD 30 in S-Tol mice (Figure 3A). Furthermore, an increase in graft-infiltrating 2W:I-A^b^ Tconvs was observed in S-Tol compared with N-Tol recipients on POD 30 (Figure 3B). At POD 60 when there was significant divergence in the allograft palpation scores in S-Tol recipients, the numbers of circulating 2W:I-A^b^ Tconvs recovered per mouse correlated with graft palpation scores (Figure 3C). In contrast, the number of 2W:I-A^b^ Tregs recovered did not correlate with tolerance (Figure 3, D–F). Finally, the percentage of 2W:I-A^b^ Tregs significantly increased in N-Tol, but not in S-Tol (Figure 3, G and H), recipients, and decreased circulating 2W:I-A^b^ Treg percentages strongly correlated with poorer graft function in S-Tol recipients (Figure 3I). Notably, there were no significant differences in the total number of circulating or graft-infiltrating Tconvs or Tregs between N-Tol and S-Tol recipients (Supplemental Figures 3 and 4). Thus, low 2W:I-A^b^ Tconv/Treg ratios systemically and within the allograft were the best biomarkers of unstable tolerance.

### Sensitization to donor OVA or Cre antigen results in linked sensitization of naive 2W:I-A^b^ Tconvs.

**Figure 4:**
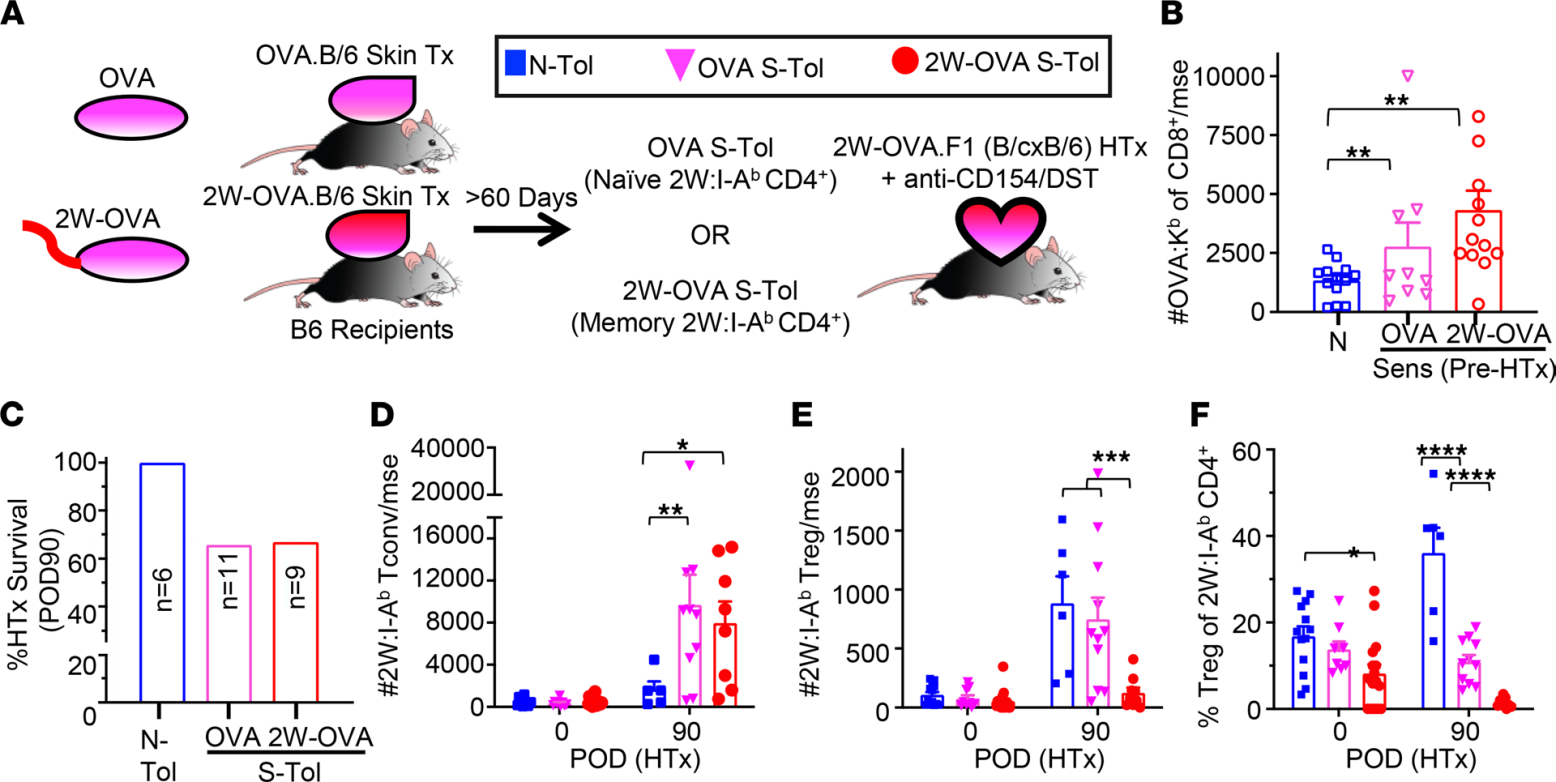
Presensitization to donor OVA antigen results in linked sensitization of naive 2W:I-A^b^ Tconvs. (**A**) Experimental design. B6 female mice were sensitized to skin grafts from female OVA.B6 or 2W-OVA.B6 donors. After 60–90 days, mice sensitized to OVA (OVA S-Tol), 2W-OVA (2W-OVA S-Tol) or naive mice (N-Tol) were transplanted with a heart graft from a 2W-OVA.F1 donor and received anti-CD154+DST. (**B**) Total number of OVA:K^b^ CD8^+^ T cells recovered (at least *n* = 9 per group) before heart transplant (POD 0). (**C**) Heart graft survival at POD 90. (**D**) Number of 2W:I-A^b^ CD4^+^ Tconvs and (**E**) Tregs and (**F**) percentage Tregs of 2W:I-A^b^ T cells from the spleen + lymph nodes on HTx POD 0 and POD 90. Each symbol represents a single mouse, and each experiment was repeated 2–3 times (*n* = 6–16 mice per group). Data are presented as mean ± STDEV, and statistical significance was assessed by 2-way ANOVA and Tukey’s or Dunnett’s multiple comparison. *P < 0.05; **P < 0.005; ***P < 0.001; ****P < 0.0001.

Two possible mechanisms can explain the induction of unstable tolerance: that erosion of graft acceptance is mediated by the accumulating memory 2W:I-A^b^ Tconvs or that memory Tconvs confer “linked sensitization” to naive BALB/c-reactive Tconvs, recruiting them to contribute to unstable tolerance. To test these possibilities, we modified our experimental model such that S-Tol mice were presensitized only to OVA via OVA.B6 skin grafts prior to receiving 2W-OVA.F1 HTx and TolRx (Figure 4A). In this case, OVA-specific CD4^+^ and CD8^+^ T cells are antigen experienced, but 2W:I-A^b^ CD4^+^ T cells are not; only OVA-specific CD8^+^ T cells can be tracked, whereas OVA:I-A^b^ tetramers do not reliably track OVA-specific CD4^+^ T cells (56). Mice sensitized by OVA.B6 or 2W-OVA.B6 skin grafts had expanded numbers of OVA:K^b^ CD8^+^ T cells (Figure 4B). By HTx POD 90, approximately 35% of OVA-S-Tol recipients had rejected their HTx (Figure 4C), thus confirming that unstable tolerance developed in these recipients.

We next tested whether naive 2W:I-A^b^ CD4^+^ T cells in OVA-S-Tol recipients followed a fate similar to that of N-Tol or 2W-OVA-S-Tol recipients. While comparable numbers of 2W:I-A^b^ Tconvs were detected between naive and OVA skin-sensitized mice, by HTx POD 90, their numbers in OVA-S-Tol recipients were significantly higher than in N-Tol recipients and comparable to those in 2W-OVA-S-Tol recipients (Figure 4D). In contrast, 2W:I-A^b^ Tregs in OVA-S-Tol recipients expanded comparably as in N-Tol and significantly more than in 2W-OVA-S-Tol. Thus, in OVA-sensitized recipients, 2W:I-A^b^ Tregs behaved like naive Tregs (Figure 4E), whereas 2W:I-A^b^ Tconvs behaved like memory Tconvs even when recipients had been sensitized only to OVA and not to 2W. As a consequence, the percentage of 2W:I-A^b^ Tregs was higher than in 2W-OVA-S-Tol but lower than N-Tol (Figure 4F).

**Figure 5:**
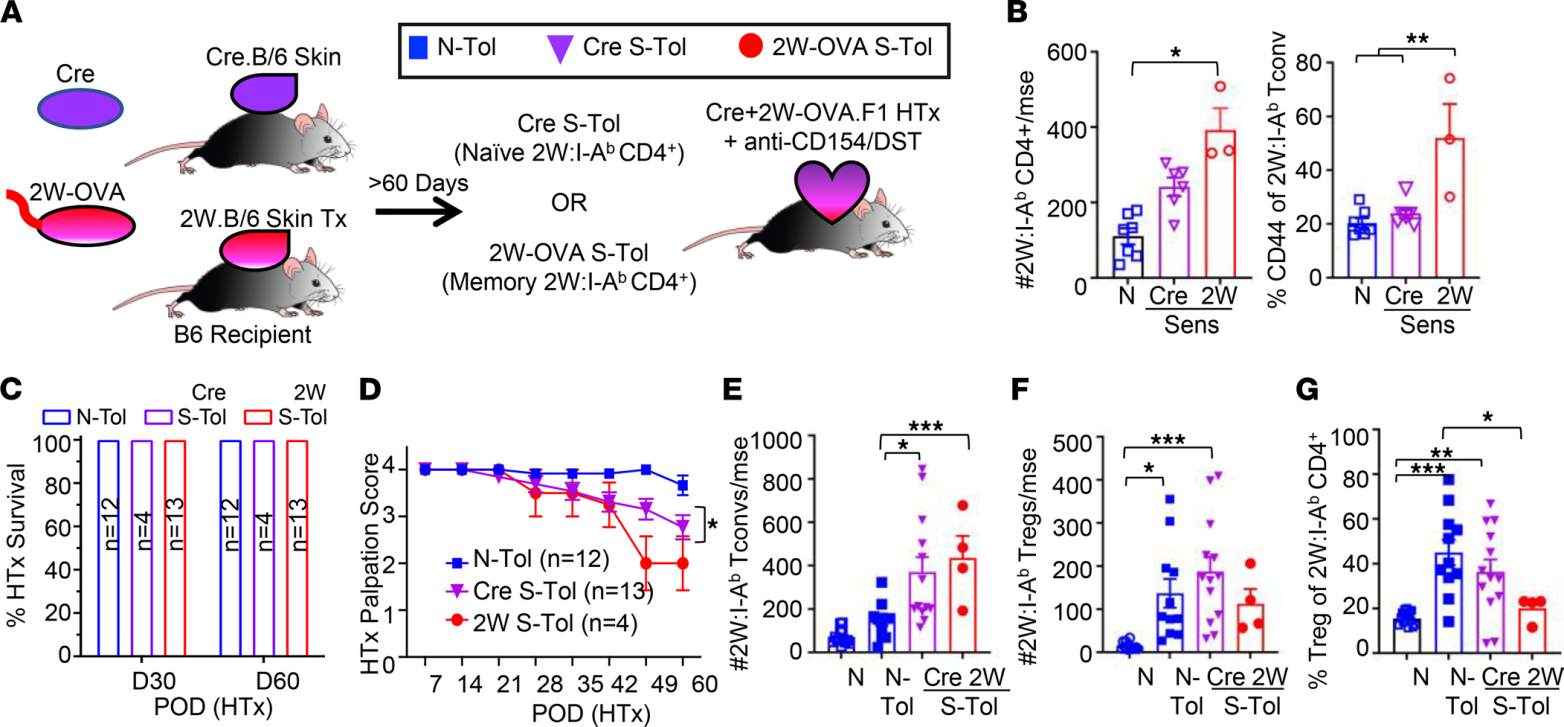
Presensitization to donor Cre antigen results in linked sensitization of naive 2W:I-A^b^ Tconvs. (**A**) Experimental design. B6 female mice were sensitized to Cre.B6 or 2W-OVA skin grafts, and after 60–90 days, were transplanted with Cre+2W-OVA.F1 hearts and received anti-CD154/DST (Cre-S-Tol and 2W-OVA-S-Tol, respectively). Naïve mice receiving Cre+2W-OVA.F1 hearts were included as controls (N-Tol). (**B**) Number of 2W:I-A^b^ CD4^+^ T cells recovered from spleen + lymph nodes per mouse before heart transplant (POD 0) and percentage of 2W:I-A^b^ CD4^+^ T cells expressing CD44. (*n* = 3–7 mice per group.) (**C**) Heart graft survival on POD 30 and POD 60. (**D**) Heart graft palpation score in N-Tol, 2W-OVA-S-Tol, and Cre-S-Tol recipients through POD 60. (**E**) Number of 2W:I-A^b^ Tconvs, (**F**) Tregs, and (**G**) percentage Tregs of 2W:I-A^b^ CD4^+^ T cells recovered from spleen + lymph nodes per mouse on POD 45–60. Each symbol represents a single mouse, and each experiment was repeated 2–3 times (*n* = 3–7 mice per group). Data are presented as mean ± STDEV, and statistical significance was assessed by 1-way ANOVA and Tukey’s or repeated measures ANOVA. *P < 0.05; **P < 0.005; ***P < 0.001.

To test whether linked sensitization would also occur with antigens expressed by the heart allograft but not physically linked to 2W-OVA, we again modified the experimental setup to sensitize mice with a Cre-expressing skin graft followed by Cre+2W-OVA.F1 HTx that coexpressed Cre and 2W-OVA antigens (Figure 5A). We confirmed that the total number of 2W:I-A^b^ Tconvs recovered was comparable in naive and Cre-sensitized mice and maintained a naive CD44^lo^ phenotype, in contrast to the elevated frequencies of CD44^hi^ 2W:I-A^b^ Tconvs observed in 2W-OVA–sensitized control mice (Figure 5B). Cre-S-Tol recipients and 2W-S-Tol recipients exhibited unstable tolerance with reduced palpation scores by HTx POD 40–60 (Figure 5, C and D), and the total number of 2W:I-A^b^ Tconvs in Cre-S-Tol recipients was significantly higher than in N-Tol recipients and was again comparable to 2W-OVA-S-Tol recipients (Figure 5D). Thus, naive 2W:I-A^b^ Tconvs in the Cre-S-Tol recipients also acquired resistance to TolRx-induced tolerance. Similar to OVA-S-Tol, 2W:I-A^b^ Tregs in Cre S-Tol recipients behaved like naive Tregs in their ability to accumulate post-HTx+TolRx (Figure 5E); as a consequence, the percentage of Tregs among 2W:I-A^b^-reactive T cells was intermediate between N-Tol and 2W-OVA-S-Tol recipients (Figure 5, F and G). Collectively, these observations suggest that linked sensitization occurs for naive Tconvs recognizing antigens coexpressed on donor grafts and raise the possibility that these cells contribute to unstable tolerance. In contrast, donor-specific Tregs were not susceptible to linked sensitization; however, the accumulation of Tregs alone was not sufficient to attain the high donor-specific Treg frequencies associated with stable tolerance.

### Naive Tconvs subject to linked sensitization acquire a phenotype of memory Tconvs.

**Figure 6:**
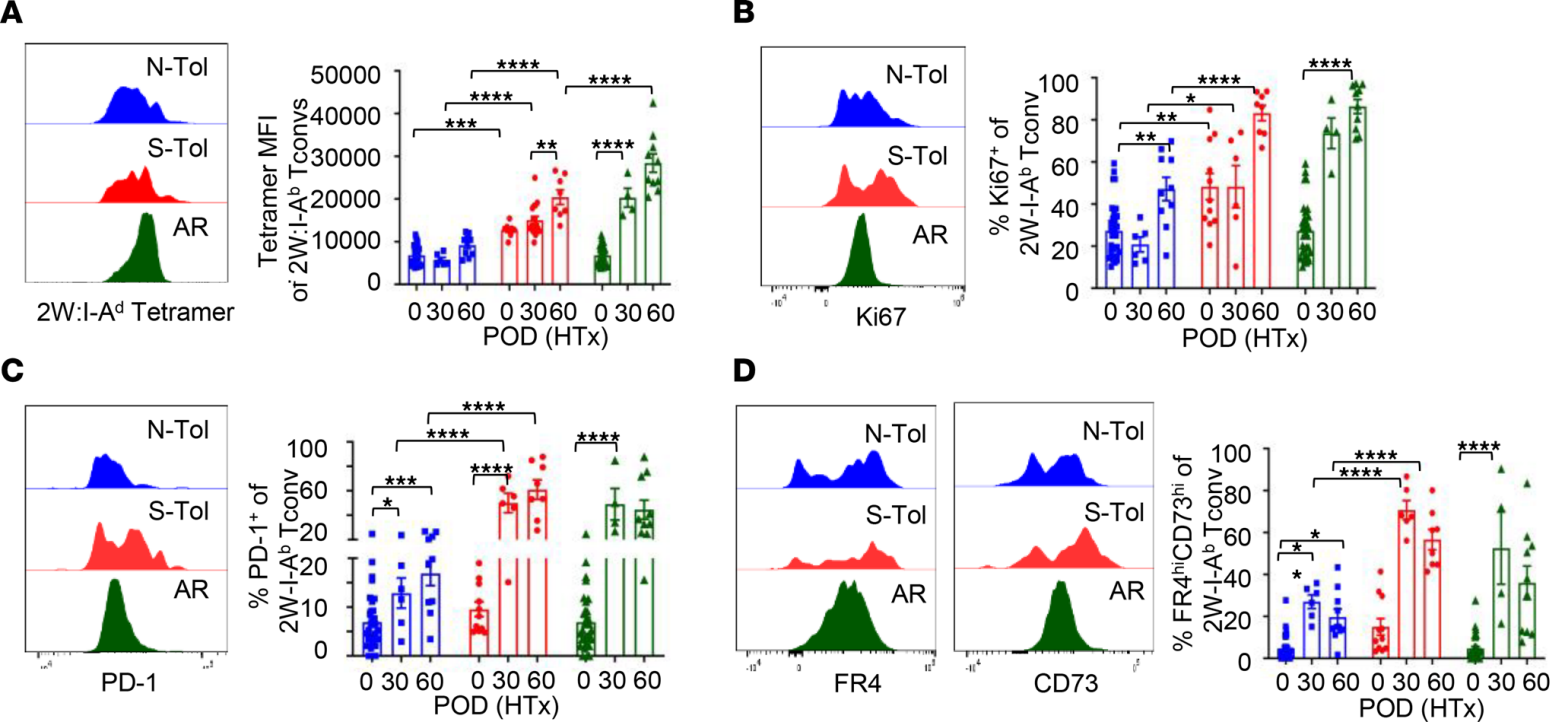
Memory Tconvs resistant to CoB-induced tolerance exhibit increased TCR avidity, as well as increased expression of proliferation and chronic activation markers. (**A**–**D**) Phenotype of 2W:I-A^b^ Tconvs from 2W-OVA skin-sensitized (S-Tol) or naive (N-Tol) mice after 2W-OVA.F1 HTx + anti-CD154+DST was assessed on POD 0, 30, and 60 (*n* = 4–32 mice per group). (**A**) Representative histogram (HTx POD 60) and MFI of 2W:I-A^b^ tetramer binding to 2W:I-A^b^ Tconvs. Representative histogram and percentage 2W:I-A^b^ Tconvs expressing (**B**) Ki-67^hi^, (**C**) PD-1^+^, and (**D**) FR4^hi^CD73^hi^. Each symbol represents a single mouse, and each experiment was repeated 2–3 times. Data are presented as mean ± STDEV, and statistical significance was assessed by 2-way ANOVA and Tukey’s multiple comparisons *P < 0.05, **P < 0.005, ***P < 0.001, ****P < 0.0001.

Donor-specific CD4^+^ Tconvs in tolerant recipients display a distinct phenotype, including low tetramer-binding avidity, reduced proliferation, and increased expression of coinhibitory molecules programmed cell death 1 (PD-1), folate receptor 4 (FR4), and CD73 (50, 57, 58). We showed that Tconvs from naive and N-Tol retained low 2W:I-A^b^ tetramer-binding avidities, whereas memory Tconvs from 2W-OVA-S-Tol recipients started with higher 2W:I-A^b^ tetramer binding prior to HTx, which further increased post-HTx (Figure 6A). The expression of Ki67, PD-1, FR4 and CD73 was also higher in S-Tol compared with N-Tol and skin-sensitized recipients (Figure 6, B–D).

**Figure 7:**
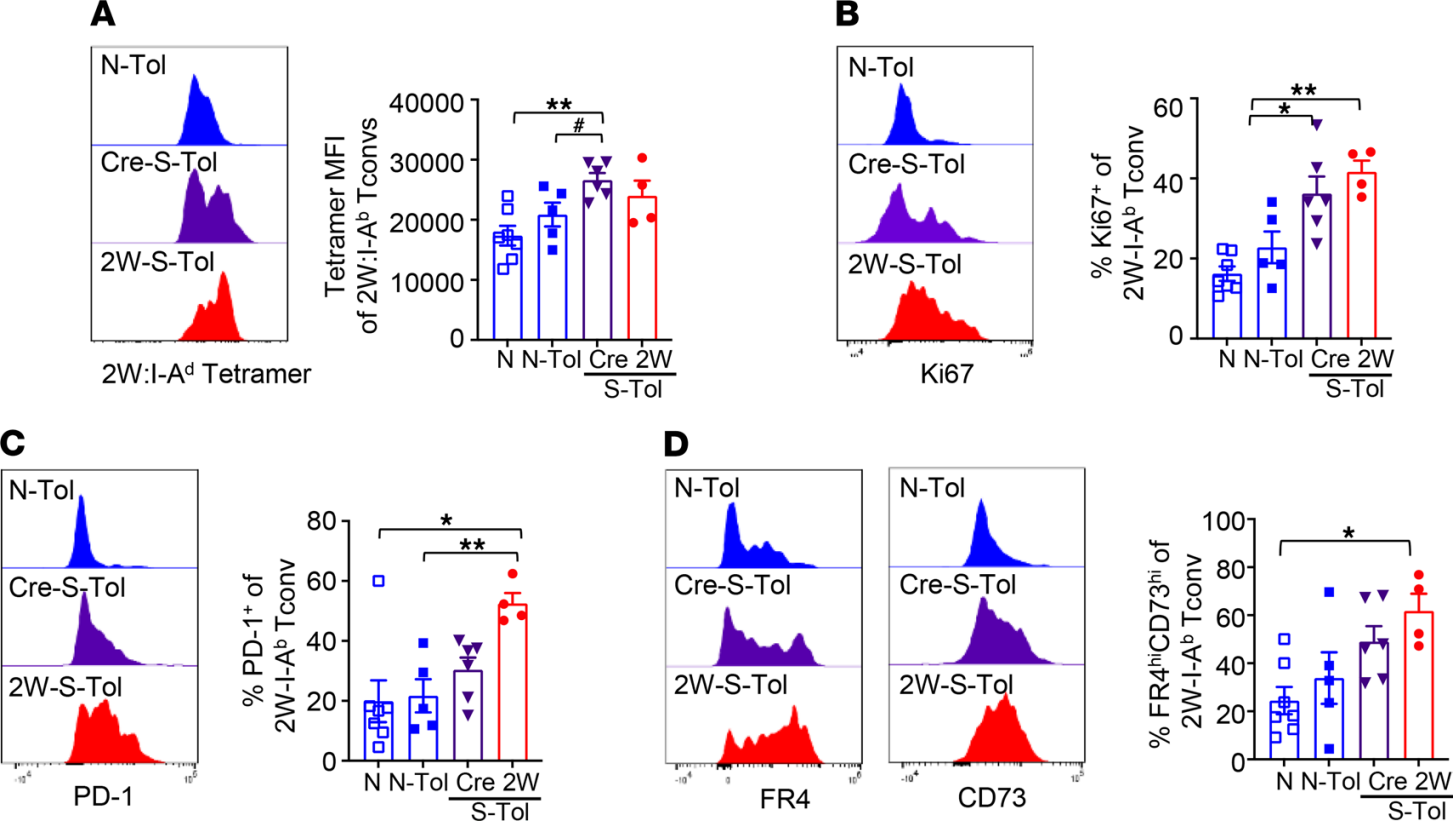
Naive Tconvs subject to infectious sensitization acquire a phenotype resembling memory Tconvs in unstable tolerance. (**A**–**D**) Phenotype of 2W:I-A^b^ Tconvs from Cre (Cre S-Tol) or 2W-OVA (2W S-Tol) skin-sensitized, or naive (N-Tol), mice, after 2W-OVA.F1 HTx + anti-CD154/DST, was assessed on HTx POD 45–60. (**A**) Representative histogram and MFI of 2W:I-A^b^ tetramer binding to 2W:I-A^b^ Tconvs. Representative histogram and percentage 2W:I-A^b^ Tconvs expressing (**B**) Ki-67^hi^, (**C**) PD-1^+^, and (**D**) FR4^hi^CD73^hi^ (*n* = 4–7 mice per group). Each symbol represents a single mouse, and each experiment was repeated 2–3 times. Data are presented as mean ± STDEV, and statistical significance was assessed by 1-way ANOVA and Tukey’s multiple comparisons *P < 0.05, **P < 0.005, or Mann-Whitney test ^#^*P* < 0.05.

Similar trends were observed in 2W:I-A^b^ Tconvs that started as naive in 2W-OVA-S-Tol recipients (Figure 7). Tetramer binding and Ki67 expression were significantly increased in 2W:I-A^b^ Tconvs from Cre-S-Tol compared with N-Tol, and percentages of 2W:I-A^b^ Tconvs expressing PD-1, FR4, and CD73 were intermediate between N-Tol and 2W-OVA-S-Tol recipients. Thus, naive donor-specific Tconvs acquired features similar to memory Tconvs of increased TCR avidity, enhanced proliferation, and expression of chronic activation markers during the development of unstable tolerance. Collectively, these observations show that donor-specific Tconvs in stable tolerance have a phenotype distinct from those in unstable tolerance. Furthermore, high expression of coinhibitory molecules is not sufficient to constrain donor-specific T cells to facilitate stable tolerance, underscoring the limits of coinhibitory molecules and highlighting the necessity to limit activation signals such as those mediated by higher-avidity TCR and proinflammatory signals.

## Discussion

Operational transplantation tolerance following the weaning of immunosuppression is achievable in the clinic, but even in the most highly selected liver transplant recipients, 40%–50% fail to achieve stable tolerance and instead develop graft rejection during or after weaning (1–9). A high frequency of memory alloreactive T cells, generated by allograft sensitization or heterologous immunity, is a key barrier to the successful induction of stable transplantation tolerance by CoB-based strategies (20–27). In this study, we tested whether a limited repertoire of memory T and B cells directed at a single donor antigen could be stably controlled by mechanisms of infectious tolerance and linked suppression. We show that mice presensitized only to the 2W-OVA antigen failed to achieve robust tolerance to 2W-OVA.F1 HTx when treated with CoB+DST and exhibited a gradual decline in cardiac function starting at POD 30, with about 33% of grafts rejecting at POD 90. Since the half-life of anti-CD154 is about 10.4 days (59), this gradual decline in graft function with immunosuppression withdrawal is reminiscent of liver transplant recipients undergoing immunosuppression withdrawal, where 7 of 20 pediatric recipients developed rejection after initiation of weaning (*n* = 4) or after complete cessation of immunosuppression (*n* = 3) (14), and approximately 60% of adult recipients experienced mild acute rejection approximately 6 months after initiation of weaning (10). Thus, we reason that this model of unstable tolerance is clinically relevant and useful for identifying mechanisms driving the resistance to stable CoB-induced tolerance.

**Figure 8:**
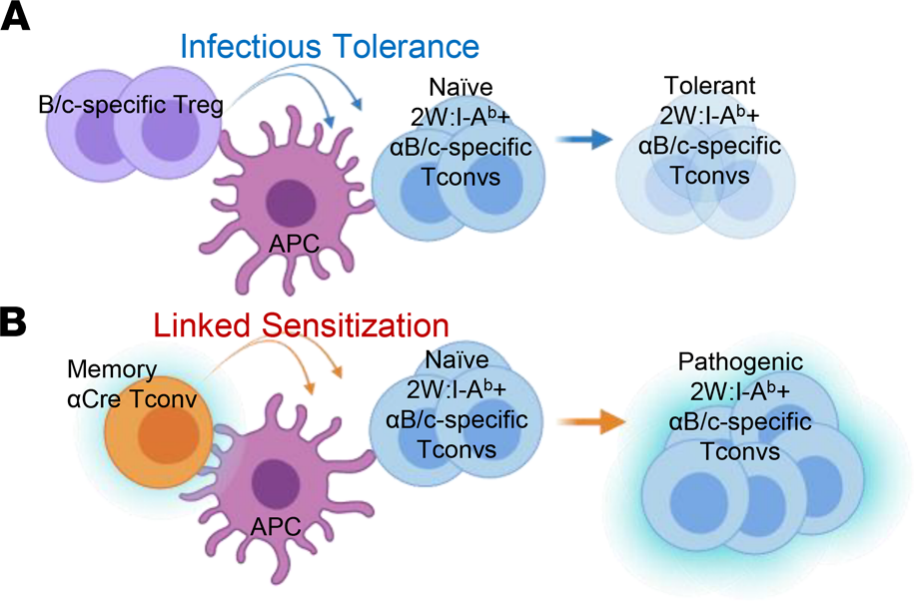
Proposed model of how linked sensitization may overcome infectious tolerance to mediate unstable tolerance. (**A**) Infectious tolerance is mediated by donor-specific Tregs through cognate interactions with antigen-presenting cells (APCs) and through bystander effects on donor-specific CD4^+^ and CD8^+^ T cells. (**B**) Linked sensitization is hypothesized to be mediated by memory T cells interacting with APCs, inducing their activation and conferring a resistance in naive donor-specific Tconvs to CoB-induced infectious tolerance mechanisms.

In recipients developing unstable tolerance, recall 2W-OVA and primary BALB/c-specific IgG responses were controlled, and memory CD8^+^ T cell expansion was inhibited, whereas memory 2W:I-A^b^ Tconvs started accumulating as early as HTx POD 30. Since donor-specific Tconvs with intrinsically reduced ability to expand are key to CoB-induced stable transplant tolerance (57, 58, 60), we conclude that the expanding number of memory Tconvs is the most likely driver of unstable tolerance. Furthermore, we demonstrate that donor-specific Tconvs that were naive at the time of tolerance induction also expanded during the development of unstable tolerance. We speculate that this phenomenon may be analogous to epitope spreading, where the immune response diversifies from an initial single epitope to subdominant and/or cryptic epitopes either on that same antigen (intramolecular spreading) or on other proteins (intermolecular spreading) and plays an active role in ongoing disease pathology (61). We named this phenomenon “linked sensitization” as an analogy to the phenomenon of linked suppression whereby tolerance can spread to new antigens through linked recognition (62). Similar to linked suppression, we hypothesize a mechanistic model in which linked sensitization is mediated by memory and naive Tconvs interacting with a common antigen-presenting cell, and where the proactivating effects of memory Tconvs eventually dominate over the protolerogenic effects of CoB on naive Tconvs (Figure 8). This phenomenon of linked sensitization by CD4^+^ Tconvs expands on the phenomenon of “incognito” CD8^+^ memory T cells previously described by Gill and colleagues to be mediating resistance to CoB-induced tolerance to allogeneic islet grafts (63). In those studies, they hypothesized that heterologous memory CD8^+^ T cells, primed by infection or environmental antigen and cross-reactive to donor antigen, would prevent the development of tolerance to the allograft. In both instances of incognito CD8^+^ memory and linked suppression, memory to a single donor antigen does not immediately license the activation of donor-reactive Tconvs. Instead, the escalating proinflammatory signals generated by a limited repertoire of donor-specific memory Tconvs eventually overcome infectious tolerance induced by CoB.

We previously reported that tolerant 2W:I-A^b^ T cells retain a low tetramer-binding avidity profile that is comparable to naive T cells, whereas rejection results in the preferential expansion of high-avidity clones that display ~3- to 4-fold higher 2W:I-A^b^ tetramer binding (50). In parallel, monoclonal alloreactive TCR75 cells in tolerant recipients develop dysfunction and upregulate expression of the coinhibitory molecule PD-1, with reduced ability to proliferate even upon adoptive transfer into secondary hosts (58). In keeping with these reports (50), we observed that 2W:I-A^b^ Tconvs did not undergo avidity maturation during stable tolerance and displayed modest increases in PD-1, FR4, and CD73, consistent with exhausted and anergic T cell phenotypes (64). In contrast, memory 2W:I-A^b^ Tconvs in unstable tolerance underwent avidity maturation and expressed high levels of Ki67, PD-1, FR4, and CD73, similar to those from recipients that had acutely rejected 2W-OVA.F1 grafts. Importantly, naive Tconvs that acquired resistance to CoB through linked sensitization also developed a similar phenotype to Tconvs that started out as memory T cells. Thus, increased coinhibitor expression on Tconvs does not invariably equate to cell dysfunction and stable tolerance but instead may indicate chronic activation leading to unstable tolerance. The activation signals that override these high levels of coinhibition require further definition, but we speculate roles for higher-avidity TCRs and proinflammatory signals generated by the memory Tconvs.

Tregs are critical mediators of transplantation tolerance (65–68). Early in the post-HTx period (POD 30), we observed a modest expansion of 2W:I-A^b^ Tregs in N-Tol but not in 2W-OVA S-Tol recipients. Furthermore, in recipients sensitized to a different epitope, OVA or Cre, 2W:I-A^b^ Tregs expanded comparably to naive Tregs, indicating that they were not susceptible to linked sensitization. One possible explanation for their reduced accumulation in 2W-OVA S-Tol recipients is that conversion of Tconvs into Tregs contributes to overall Treg accumulation in N-Tol recipients, while the conversion of memory Tconvs into induced Tregs is impaired in 2W-OVA S-Tol recipients. Consistent with previous reports (39, 69), adoptively transferred, congenically labeled Tconvs sorted from Foxp3-GFP reporter mice converted into Foxp3^+^ Tregs that preferentially accumulated in allografts of N-Tol recipients (Supplemental Figure 6, A and B). Unfortunately, the frequencies of 2W:I-A^b^ Tregs (<0–20/graft) in these mice were too low to reliably test whether memory Tregs had reduced conversion compared with naive Tregs. Using flow-sorted naive (CD44^–^) and memory (CD44^+^) TCR75 cells, we demonstrated that the in vitro rates of conversion in the presence of TGF-β (70) of flow-sorted memory Tconvs were significantly reduced compared with naive Tconvs (Supplemental Figure 6C). Finally, the manner in which Tregs mediate transplantation tolerance remains unclear but likely occurs through multiple mechanisms (71). We show that donor-specific Treg numbers alone did not discriminate between stable and unstable tolerance or rejection; instead, high donor-specific Treg/Tconv ratios were more instructive. These observations underscore the necessity of controlling donor-specific Tconv expansion to achieve stable transplant tolerance and raise the possibility that adoptive transfer of donor-specific Tregs may be able to promote stable transplant tolerance.

In summary, we show that pretransplant memory to a single donor antigen is sufficient to prevent the induction of stable tolerance by CoB. While the expansion of memory CD8^+^ T cells and recall 2W-OVA IgG were controlled in the recipients developing unstable tolerance, memory Tconvs accumulated and acquired multiple features resembling Tconvs from acute rejecting recipients. We also show that through linked sensitization, naive donor-specific Tconvs acquired a phenotype resembling memory Tconvs. The observations that linked sensitization can override infectious tolerance highlight the major barrier posed by memory to even a single donor antigen and emphasize the necessity of identifying mechanisms for inducing a cell-intrinsic dysfunctional state in memory Tconvs in order to achieve stable transplant tolerance.

## Methods

### Mice.

Female B6 (H-2^b^) and BALB/c (H-2^d^) mice, ages 4–9 weeks, were purchased from The Jackson Laboratory or Harlan Laboratories. B6.C-Tg(CMV-cre)1Cgn/J and C57BL/6-Tg(CAG-OVAL)916Jen/J mice, ages 8–9 weeks, were purchased from The Jackson Laboratory. B6 were bred with 2W-OVA.BALB/c mice to obtain 2W-OVA.F1 mice, and B6.C-Tg(CMV-cre)1Cgn/J mice were bred with 2W-OVA.BALB/c mice to obtain Cre+2W-OVA.F1 mice. TCR75 TCR-Tg mice were obtained from R. Pat Bucy (University of Alabama at Birmingham, Birmingham, Alabama, USA) and crossed to CD45.1 mice in the animal facilities of the University of Chicago. Foxp3-GFP C57BL/6 mice were purchased from The Jackson Laboratory and crossed to CD45.1 mice in the animal facilities of the University of Chicago.

### Sensitization by skin transplantation.

OVA^+^ or 2W-OVA^+^ or Cre^+^ mice were sacrificed, and their skins were cut into ~0.5 cm^2^ sections and grafted onto the flanks of recipient B6 mice. Skin grafts were completely rejected by 30 days posttransplant across all models and after about 60 days were used for heart transplant recipients.

### Heart transplantation and tolerance induction.

Heterotopic heart transplantations were performed as previously described (72), by implanting hearts from 6- to 8-week-old donors into the peritoneal cavity of B6 recipients by end-to-side anastomosis of the donor aorta with recipient aorta and the donor pulmonary artery with recipient inferior vena cava. Each mouse received intravenous anti-CD154 (500 μg/mouse; MR1 (BioXCell) together with 2.0 × 10^7^ to 2.5 × 10^7^ donor splenocytes on the day of transplant, followed by 2 additional doses of 250 μg/mouse anti-C154 on days 7 and 14 posttransplant. Heart grafts were measured for survival by abdominal palpation. Palpation scoring was as follows: 4: strong and fast heartbeat, small heart; 3: some slowing of heartbeat or graft enlargement; 2: significant slowing of heartbeat; 1: faint and/or slow heartbeat; 0: undetectable heartbeat.

### Tissue processing for flow cytometry.

Spleens and inguinal, axial, and brachial lymph nodes were harvested and passed through a 40 μM strainer (Corning, catalog 431750). Heart tissue for flow cytometry analysis was cut into approximately 2  mm^3^ pieces in HBSS (Gibco, Thermo Fisher Scientific) and incubated for 20 minutes at 37°C with collagenase II (MilliporeSigma), DNaseI (Roche), and HEPES (Gibco, Thermo Fisher Scientific) prior to passing through a 70 μM strainer.

Lymphocytes were enriched for either CD4^+^ (Miltenyi Biotec, catalog 130-104-454) cells or pan-T cells by negative selection (Miltenyi Biotec, catalog 130-095-130) prior to staining to maximize the number of tetramer-specific cells detected. Samples were stained for flow cytometry using LiveDead Aqua (Invitrogen, Thermo Fisher Scientific) or LiveDead NearIR (Invitrogen, Thermo Fisher Scientific) to exclude dead cells. An antibody cocktail was used to exclude unwanted cells, consisting of CD49b (DX5, catalog 485971-82, Invitrogen, Thermo Fisher Scientific), CD11c (N418, catalog 48-0114-82), F4/80 (BM8, catalog 48-4801-82, Invitrogen, Thermo Fisher Scientific), NK1.1 (PK136, catalog 48-5941-82 eBioscience, Thermo Fisher Scientific), Ter-119 (Ter-119, catalog 48-5921-82, eBioscience, Thermo Fisher Scientific), and CD19 (eBio1D3, catalog 48-0193-82, Invitrogen, Thermo Fisher Scientific), CD4 (RM4-5, catalog 48-0042-82, eBioscience, Thermo Fisher Scientific), or CD8 (53-6.7, catalog 612898, BD Biosciences), depending on the experiment. Additional antibodies against CD90.2 (53-2.1, 565257, BD Biosciences), CD4 (GK1.5, 612952, BD Biosciences), CD44 (IM7, catalog 560570, BD Biosciences), CD62L (MEL-14, catalog 563252, BD Biosciences), Ki67 (B56, catalog 561283, BD Biosciences), PD-1 (RMP1-30, catalog 109110, BD BioLegend), CD73 (TY/11.8, catalog 127215, BioLegend), CD25 (PC61, catalog 102015, BioLegend), CTLA-4 (UC10-4F10-11, catalog 565778, BD Biosciences), T-bet (O4-46, catalog 564141, BD Biosciences), CD223 (C9B7W, catalog 751318, BD Biosciences), TIGIT (1G9, catalog 744213, BD Biosciences), CD127 (SB/199, catalog 612841, BD Biosciences), FR4 (eBio12A5, catalog 25-5445-82, eBioscience, Thermo Fisher Scientific), CD73 (TY/11.8, catalog 127215, BioLegend), CD154 (MR1, catalog 740685, BD Biosciences), GITR (DTA-1, catalog 126315, BioLegend), CD39 (24DMS1, catalog 46-0391-82, Invitrogen, Thermo Fisher Scientific), and Foxp3 (FJK-16s, catalog 53-5773-82, Invitrogen, Thermo Fisher Scientific) were used to stain T cells. All PE- and APC-conjugated tetramers were obtained from the NIH Tetramer Facility: 2W (EAWGALANWAVDSA):I-A^b^ tetramers and OVA (SIINFEKL):H-2K^b^ tetramer incubation was performed at room temperature for 40 minutes prior to addition of other antibodies. The LSR Fortessa (BD) or Cytek Aurora (Cytek Biosciences) was used to quantify the flow cytometry samples.

### Donor-specific antibody quantification.

To determine titers of donor-specific antibodies in the serum of recipients, 1 × 10^6^ BALB/c or 2W.OVA.B6 splenocytes were incubated for 30 minutes at 4°C with 5 μL of serum from recipient mice. Cells were then washed and incubated with anti-CD19 (1D3, catalog 550992, BD Biosciences) and goat anti-mouse IgG (H+L) (catalog 1031-02, Southern Biotech) for 30 minutes at 4°C. MFI of the CD19^–^ cells that were IgG positive was measured by flow cytometry on the Cytek Aurora.

### Histology.

Heart allografts were harvested and sliced longitudinally, fixed in 10% formalin for 24 hours, and transferred to 70% ethanol for storage. Tissue was then embedded in paraffin, and sections (10 μm) were cut and stained with H&E or with anti-CD4 (catalog 14-9766-80, Invitrogen, Thermo Fisher Scientific), Foxp3 (catalog 14-5773-82, Invitrogen, Thermo Fisher Scientific), CD8 (catalog 14-0808-82, Invitrogen, Thermo Fisher Scientific), or C4d (catalog HP8033, Hycult Biotech) antibodies. Slides were then scanned using the Olympus VS200 SlideView Whole Slide Scanner at 40× original magnification with a numerical aperture of 0.95. H&E quantification was performed in a single-blind manner, and heart tissue was scored according to the amount of damage using the following scale: cellular infiltration component 0: no notable cellular infiltrate, 1: minor cellular infiltrate, 2: moderate cellular infiltrate 3: significant cellular infiltrate, 4: ubiquitous cellular infiltrate; graft structure component 0: normal graft structure, 1: minor disruptions to vessels and heart structure, 2: moderate disruptions to vessels and heart structure, 3: major disruptions to vessels and heart structure and signs of necrosis or scarification; 4: ubiquitous disruptions to vessels and heart structure and signs of necrosis or scarification. Immunohistochemistry quantification was performed using open-source QuPath software with built-in ‘‘positive cell detection’’ (73).

### In vitro cytokine stimulation and Tconv-to-Treg conversion assay.

For cytokine stimulation assay, spleen cells were harvested from transplanted mice on POD 60, and pan-T cells were enriched by negative selection (Miltenyi Biotec, catalog 130-095-130). Enriched T cells were stimulated overnight in vitro with anti-CD3+anti-CD28 or T cell–depleted, LPS-activated, 2W-OVA.F1 splenocytes. BD GolgiPlug was added to cells 6 hours before harvest. Optimized procedure for assessing the functional activity of tetramer-binding CD4^+^ T cells was based upon on the simultaneous intracellular staining with MHC tetramers and cytokine-specific antibodies (74). For Treg conversion assay, CD4^+^ T cells were negatively enriched (Miltenyi Biotec, catalog 130-104-454) from TCR75 mice. CD4^+^ T cells were stimulated for 24 hours with 1 mg/mL plate-bound anti-CD3. TGF-β (1–2.5 ng/mL) was added at the time of plating.

### Statistics.

Statistical analysis was performed using GraphPad Prism. Two-tailed unpaired *t* tests were used to calculate differences between experimental animals. One-way ANOVA with Bonferroni’s post hoc test or 2-way ANOVA with Tukey’s post hoc test for multiple comparisons or Dunnett’s multiple comparisons test, where appropriate, were performed to determine significance of differences between groups. *P* values below 0.05 were considered significant.

### Study approval.

All animal experiments were approved by the Institutional Animal Care and Use Committee at the University of Chicago and adhered to the standards of the NIH *Guide for the Care and Use of Laboratory Animals* (National Academies Press, 2011).

## Author contributions

MSA and JSY designed and performed experiments, analyzed and interpreted the data, and edited the manuscript. JMP assisted in the flow analysis and edited the manuscript, and DY performed the organ transplantations. MLA contributed to the data interpretation and edited the manuscript. and ASC conceived and oversaw the project and interpretation of the data and cowrote, with MSA, and edited the manuscript.

## Supplementary Material

Supplemental data

## Version 1 06/08/2022

Electronic publication
