# Supplementary material for: Linked sensitization by memory CD4+ T cells prevents costimulation blockade–induced transplantation tolerance
Source: JCI Insight. 2022 Jun 8;7(11):e159205. doi: 10.1172/jci.insight.159205 (PMC9220839; doi:10.1172/jci.insight.159205)
Supplement: Supplemental data [file jciinsight-7-159205-s182.pdf]

## Supplementary Figure Legends

**Fig S1.** Experimental design for sensitizing recipients with 2W-OVA-, OVA- and Cre-transgenic, **A-C,** C57BL/6 skin transplants, followed by anti-CD154/DST to induce tolerance to 2W-OVA.F1, **A-B,** or Cre-2W-OVA.F1, **C,** hearts. Tracking of 2W:I-$A^b$ $CD4^+$ or OVA:$K^b$ $CD8^+$ T cells that start as memory or naïve at the time of heart transplantation, or graft-specific IgG.

**Fig S2. A-B,** Gating strategy for OVA:$K^b$ $CD8^+$ T cells to determine phenotype and dual IFN$\gamma$/TNF$\alpha$ production. **C,** Gating strategy for 2W:I-$A^b$ $CD4^+$ Tconvs and Tregs.

**Fig S3.** Unstable tolerance is not associated with increased accumulation of CD8 $^+$T cells, $CD4^+$ Tconvs or reduced Tregs in the heart allografts. Representative immunohistochemistry staining at 40x magnification and quantification of cell number per cm2 on POD60 was conducted on a total of n=4 sections per group using QuPath automatic cell detection software **A,** CD8 $^+$T cells by histology. **B,** $CD4^+$ Tconvs by Flow Cytometry, **C,** $CD4^+$ Tconvs by histology, **D,** $CD4^+$ Tregs by Flow Cytometry **E,** $CD4^+$ Tregs by histology **F,** %Tregs in the heart allografts by flow cytometry, and **G,** reduced %Tregs in the heart allografts by histology.

**Fig S4.** Comparable recovery of **A,** all $CD4^+$ **B,** Tconvs, **C,** Tregs and **D,** % Tregs from pooled secondary lymphoid organs of stable tolerant (naïve; N-Tol) or unstable tolerant (2W-OVA-sensitized; S-Tol) recipients before heart transplant (POD0), POD30 and POD60 (at least n=6 per group).

**Fig S5.** Memory OVA:$K^b$ $CD8^+$ T cells eventually accumulate late (HTx POD90) in unstable tolerance, in recipients sensitized with skin grafts from **A,** 2W-OVA (2W Sens), **B,** Cre or 2W-OVA, **C,** OVA donors, and then received **A+C,** 2W-OVA.F1, or **B,** Cre-2W-OVA.F1 heart transplants and anti-CD154/DST. N: naïve; NT: naïve + HTx + anti-CD154+DST; ST: sensitized + HTx + anti-CD154/DST (n>4 mice per group).

**Fig S6. A,** Experimental design. **B,** Adoptively transferred Tconvs converted to $Foxp3^+$ Tregs and preferentially accumulated in the grafts of tolerant mice receiving 2W-OVA.F1 + CoB (n= 7-12 mice per group).**C,** Experimental design **D,** Flow-sorted memory $CD44^+CD4^+$ Tconvs from TCR75 TCR transgenic mice exhibit reduced conversion to Tregs in vitro when stimulated with anti-CD3 for 24 hrs in the presence of TGFß (1-2.5ng/mL) as compared to naïve $CD44^-CD4^+$ TCR75 TConvs (n = 4 mice per group).

Figure S1

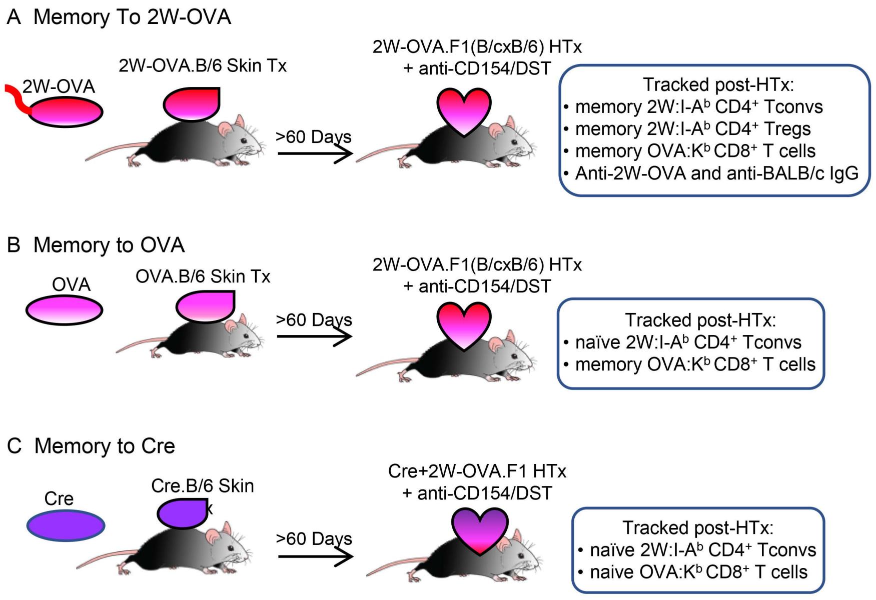
A Memory To 2W-OVA
2W-OVA
2W-OVA.B/6 Skin Tx
>60 Days
2W-OVA.F1(B/cxB/6) HTx
+ anti-CD154/DST
Tracked post-HTx:
• memory 2W:I-A$^b$ CD4$^+$ Tconvs
• memory 2W:I-A$^b$ CD4$^+$ Tregs
• memory OVA:K$^b$ CD8$^+$ T cells
• Anti-2W-OVA and anti-BALB/c IgG
B Memory to OVA
OVA
OVA.B/6 Skin Tx
>60 Days
2W-OVA.F1(B/cxB/6) HTx
+ anti-CD154/DST
Tracked post-HTx:
• naïve 2W:I-A$^b$ CD4$^+$ Tconvs
• memory OVA:K$^b$ CD8$^+$ T cells
C Memory to Cre
Cre
Cre.B/6 Skin
>60 Days
Cre+2W-OVA.F1 HTx
+ anti-CD154/DST
Tracked post-HTx:
• naïve 2W:I-A$^b$ CD4$^+$ Tconvs
• naive OVA:K$^b$ CD8$^+$ T cells

Figure S2

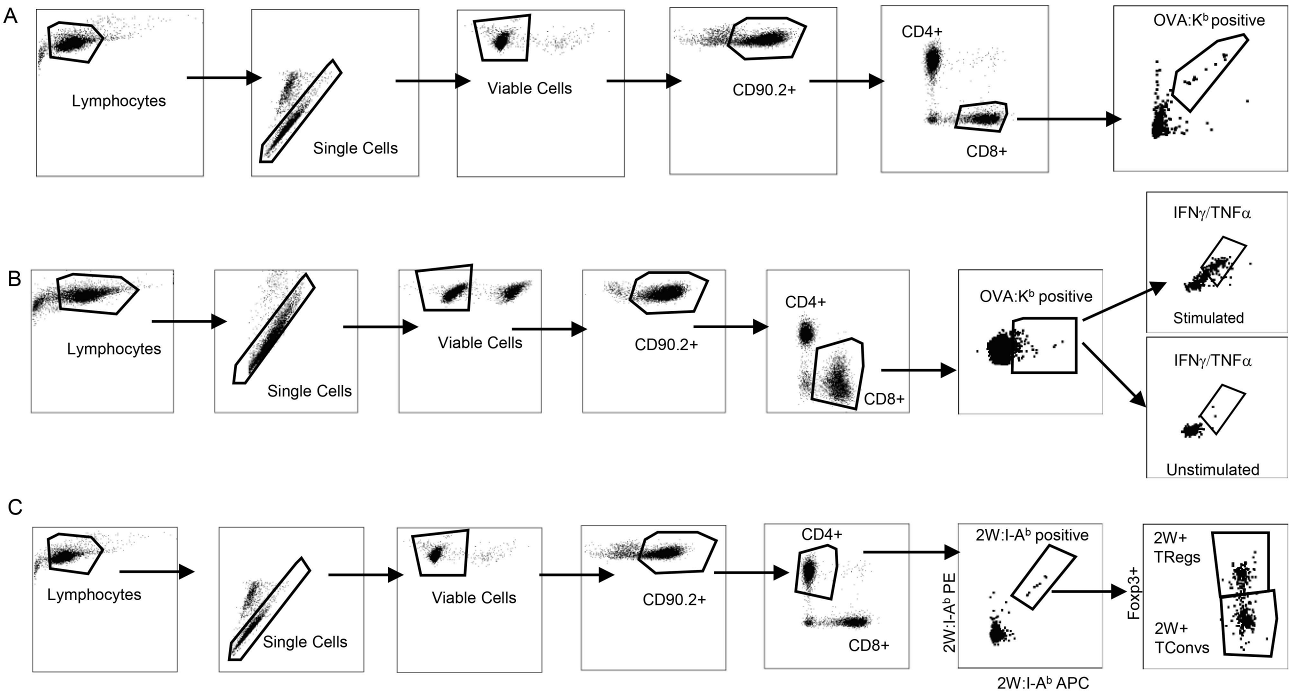
A
Lymphocytes
Single Cells
Viable Cells
CD90.2+
CD4+
CD8+
OVA:K$^b$ positive
B
Lymphocytes
Single Cells
Viable Cells
CD90.2+
CD4+
CD8+
OVA:K$^b$ positive
IFNγ/TNFα
Stimulated
IFNγ/TNFα
Unstimulated
C
Lymphocytes
Single Cells
Viable Cells
CD90.2+
CD4+
CD8+
2W:I-A$^b$ positive
2W:I-A$^b$ PE
2W:I-A$^b$ APC
Foxp3+
2W+ TRegs
2W+ TConvs

Figure S3

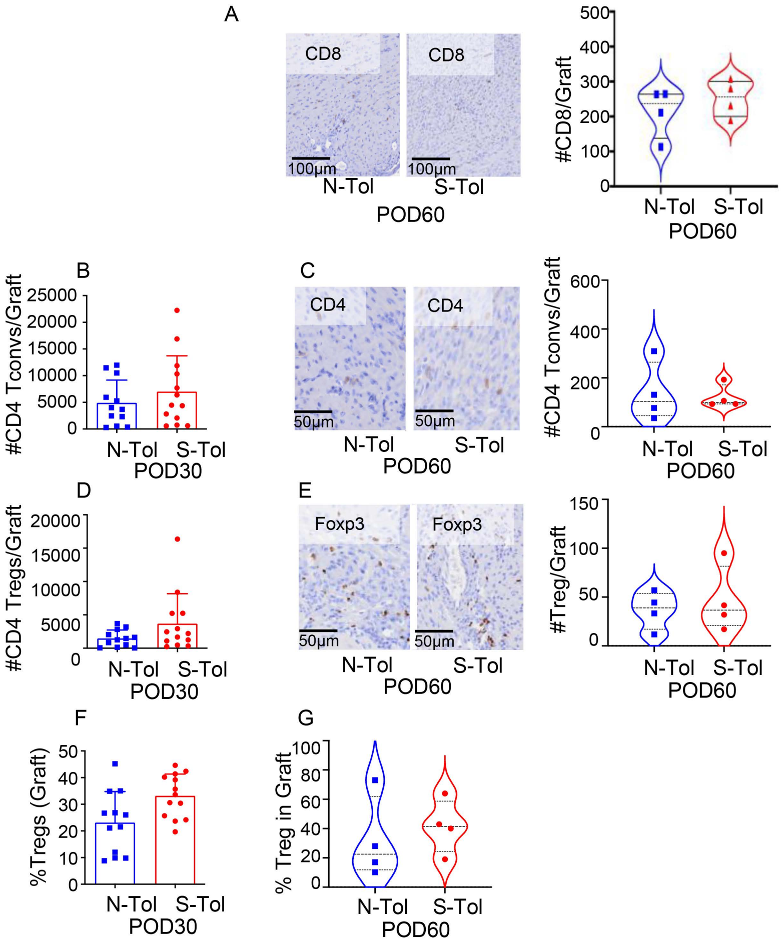
A
CD8
CD8
100μm
100μm
N-Tol
S-Tol
POD60
#CD8/Graft
500
400
300
200
100
0
N-Tol
S-Tol
POD60
B
#CD4 Tconvs/Graft
25000
20000
15000
10000
5000
0
N-Tol
S-Tol
POD30
C
CD4
CD4
50μm
50μm
N-Tol
S-Tol
POD60
#CD4 Tconvs/Graft
600
400
200
0
N-Tol
S-Tol
POD60
D
#CD4 Tregs/Graft
20000
15000
10000
5000
0
N-Tol
S-Tol
POD30
E
Foxp3
Foxp3
50μm
50μm
N-Tol
S-Tol
POD60
#Treg/Graft
150
100
50
0
N-Tol
S-Tol
POD60
F
%Tregs (Graft)
50
40
30
20
10
0
N-Tol
S-Tol
POD30
G
% Treg in Graft
100
80
60
40
20
0
N-Tol
S-Tol
POD60

Figure S4

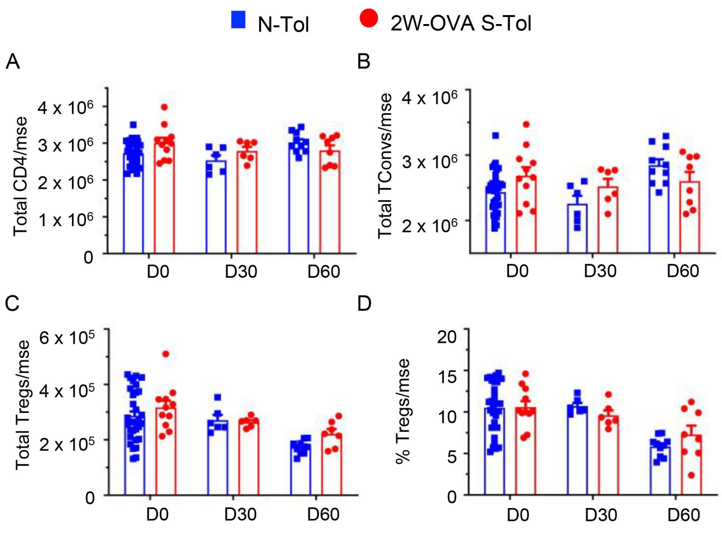
N-Tol
2W-OVA S-Tol
A
Total CD4/mse
4 x 10^6
3 x 10^6
2 x 10^6
1 x 10^6
0
D0
D30
D60
B
Total TConvs/mse
4 x 10^6
3 x 10^6
2 x 10^6
D0
D30
D60
C
Total Tregs/mse
6 x 10^5
4 x 10^5
2 x 10^5
0
D0
D30
D60
D
% Tregs/mse
20
15
10
5
0
D0
D30
D60

Figure S5

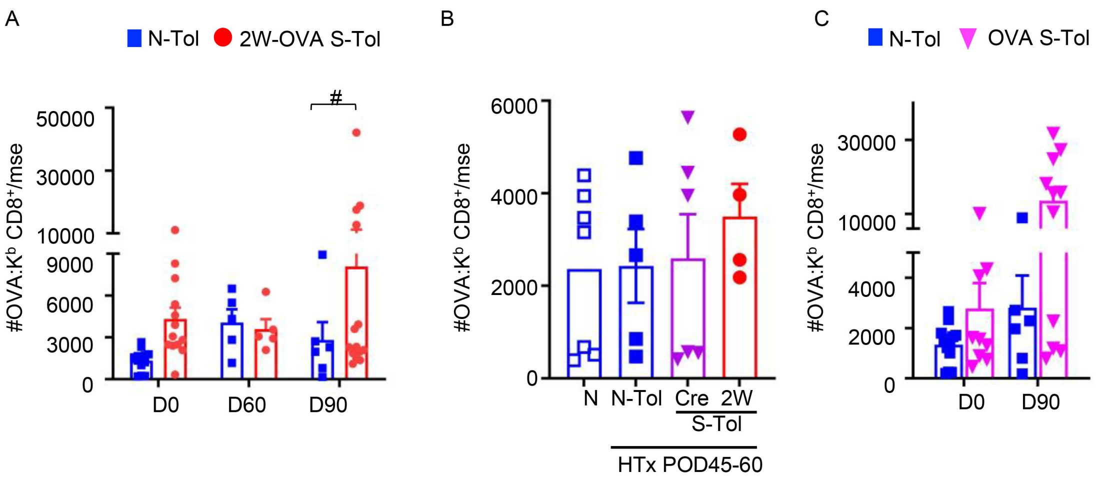

A
N-Tol
2W-OVA S-Tol
#OVA:K$^b$ CD8$^+$/mse
50000
30000
10000
9000
6000
3000
0
#
D0
D60
D90
B
6000
4000
2000
0
#OVA:K$^b$ CD8$^+$/mse
N
N-Tol
Cre
2W
S-Tol
HTx POD45-60
C
N-Tol
OVA S-Tol
#OVA:K$^b$ CD8$^+$/mse
30000
10000
4000
2000
0
D0
D90

Figure S6

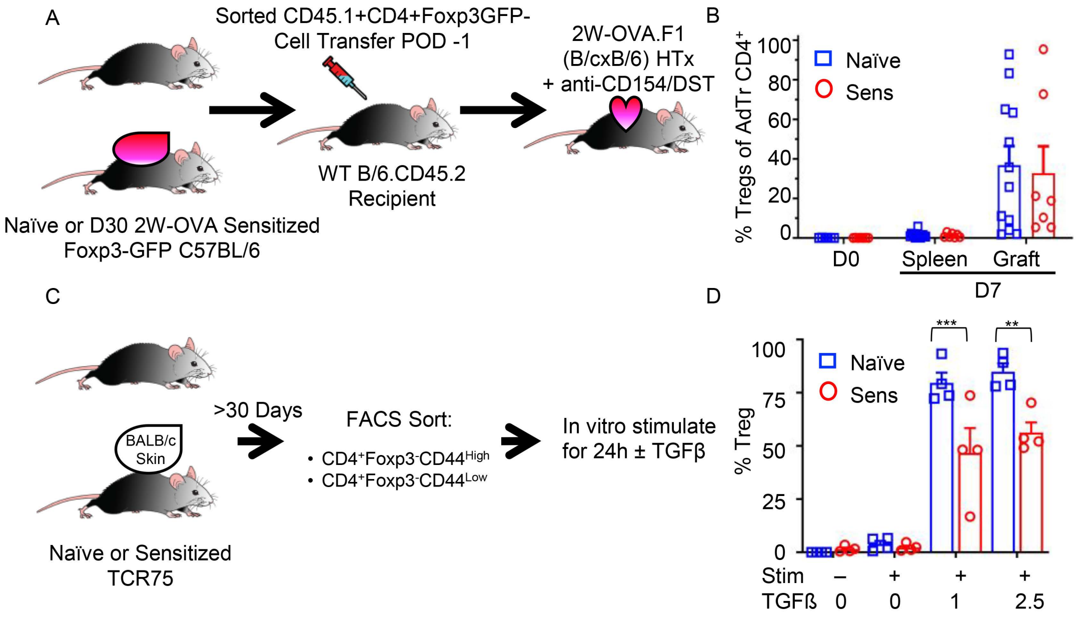
A
Sorted CD45.1+CD4+Foxp3GFP-
Cell Transfer POD -1
2W-OVA.F1
(B/cxB/6) HTx
+ anti-CD154/DST
WT B/6.CD45.2
Recipient
Naïve or D30 2W-OVA Sensitized
Foxp3-GFP C57BL/6
B
% Tregs of AdTr CD4+
100
80
60
40
20
0
Naïve
Sens
D0
Spleen
Graft
D7
C
BALB/c
Skin
Naïve or Sensitized
TCR75
>30 Days
FACS Sort:
• CD4+Foxp3-CD44High
• CD4+Foxp3-CD44Low
In vitro stimulate
for 24h ± TGFβ
D
% Treg
100
75
50
25
0
Naïve
Sens
***
**
Stim – + + +
TGFß 0 0 1 2.5
